# The Effectiveness of Self-Instructional Video vs. Classroom Teaching Method on Focused Assessment With Sonography in Trauma Among House Officers in University Hospital

**DOI:** 10.3389/fsurg.2021.698774

**Published:** 2021-08-17

**Authors:** Mohd Hisham Isa, Kristina Lim, Mohd Johar Jaafar, Ismail Mohd Saiboon

**Affiliations:** ^1^Department of Emergency Medicine, Faculty of Medicine, Universiti Kebangsaan Malaysia, Kuala Lumpur, Malaysia; ^2^Emergency and Trauma Department, Hospital Raja Permaisuri Bainun, Ipoh, Malaysia

**Keywords:** focused assessment with sonography in trauma, education, self-directed learning, simulation, self-instructional video

## Abstract

**Background:** The aim of this study was to compare the effectiveness of self-instructional-video (SIV) and classroom training method (CTM) in learning Focus-Assessment with Sonography-in-Trauma (FAST) among house officers (HO).

**Method:** A randomized controlled study involving house officers working in the university hospital in Malaysia was conducted where participants were randomized into SIV group (intervention) and CTM group (control). Each group had to undergo a 4 h hands-on training. The intervention group has undergone self-training using the video material without any facilitation while the control group received lecture and hands-on training with facilitators. Participants' performance was assessed using a validated Objective Structured Clinical Examination checklist for landmark identification and interpretation of images generated. Learning preference and confidence level were also assessed.

**Result:** A total of 33 HO were enrolled in this study. Marks obtained in image acquisition by the intervention and control were 25.3 (SD = 5.3) and 25.6 (SD = 2.3) *p* > 0.05, respectively. While in image interpretation, the mean score for the intervention and control group was 10.3 (SD 1.7) and 9.8 (SD = 1.7) *p* > 0.05, respectively. Overall performance assessment, showed the intervention group obtained 35.6 (SD = 5.9) compared to control 35.3 (SD = 3.4), *p* > 0.05. Based on pre-specified determinant these scores difference falls within the 10% of non-inferiority margin. The absolute difference between both groups was 0.3 (CI = −3.75 to 3.21, *p* = 0.871), which proves non-inferiority but not superiority. In terms of learning preference and confidence to perform FAST, most of the participants preferred the control group approach.

**Conclusion:** The SIV method is as effective as the CTM for learning FAST among the house officers and served as an alternative to classroom teaching. However, this technique needs improvement in promoting their confidence and preference. Perhaps incorporating a feedback session after going through the SIV would improve the confidence.

## Introduction

Focused Assessment with Sonography in Trauma (FAST) is a protocol used to detect hemoperitoneum and hemopericardium using ultrasonography in trauma cases (1). It has long been used as an adjunct in primary survey and even adapted in the Advanced Trauma Life Support (ATLS) algorithm since 2007 (2, 3). Although ultrasonography is not the gold standard for diagnosing intra-abdominal injury, it became popular due to its portability, ease of use and capability to be performed repeatedly without concerns of radiation exposure (4, 5). FAST is routinely done for trauma cases in the emergency department (1, 5).

Currently most ultrasound courses are conducted face-to-face in a classroom with hands-on practice. The classroom teaching method (CTM) requires a qualified instructor to deliver a didactic classroom session followed by hands-on training using patients with findings or simulated patients without findings. Incorporating simulation as a teaching modality is the way by which health educators can bridge the existing gaps (6). An ultrasound simulator may be used to conduct such training, but it is expensive. FAST usually requires long hours of training and is not easy to arrange for a large crowd, especially when time and resources are limited. Besides that, participants may be pressured to keep up with their peers' pace in a classroom teaching since it is usually done only once (7, 8). Hence some might find it challenging to fully benefit from classroom learning, especially when it involves participants from different educational backgrounds and experiences such as doctors from different specialities, nurses and paramedics. Lack of a clinical instructor to teach the skill is also another problem (9). Due to the increasing need for healthcare professional manpower, the ratio of instructor to trainees has increased, placing more burden on the instructor in terms of time and responsibility (10).

There are newer teaching modalities that has been proposed such as incorporating the use of video-assisted learning through self-instructional video (SIV) with ultrasound simulators for hands-on training to teach sonographic skills (7, 8). The impact of this method is not limited to reducing the need for the instructor's presence, but also enabling participants to review the video for learning at their own time and pace, allowing a more relaxed learning environment (10, 11). Hence, participants do not have to worry about keeping up with the instructor's or the other participants' pace during the lecture (7, 12). The impact is enhanced during the COVID-19 pandemic, when it is harder to conduct a classroom training course due to strict measures laid down by the authorities in line with lockdowns or Movement Control Orders (13). SIV is also gaining better acceptance as a tool for learning sonographic skills as evidenced by the use of videos in online courses. The technology is readily available and can be easily accessed and viewed from gadgets such as laptops and smartphones (14, 15).

Several studies have been conducted to evaluate the effectiveness of SIV in teaching clinical procedures where the main benefit lies in its flexibility (8, 10). The module is also easily repeatable and can compensate for the lack of instructor and time (8, 12). A previous study showed that an animated video can be an effective teaching tool (16). SIV has been used to teach senior doctors or specialists of certain specialties such as anaesthesiology and pediatric emergency medicine (8, 17). However, teaching sonographic procedures such as FAST via SIV to junior house officers or doctors has not been thoroughly investigated. In our study, we aim to evaluate the effectiveness of teaching FAST using SIV compared with face-to-face CTM among house officers. It is hoped that more healthcare professionals can be trained effectively to perform FAST using SIV.

## Methods

We conducted a prospective randomized interventional study in a university hospital in Kuala Lumpur, Malaysia from 1st June 2019 to 30th January 2020. We enrolled house officers from various departments in the hospital except from the emergency department. None of them had undergone formal training for FAST or abdominal ultrasonography. In this study we developed a self-directed-learning-package (SDLP) for FAST and evaluated the effectiveness of the FAST SDLP in terms of confidence and preference using a validated questionnaire. Psychomotor skills were evaluated using Objective Structured Clinical Examination (OSCE).

### The Intervention

#### Development of Self-Directed Learning Package

The SDLP consists of a video lecture and a self-instructional video (SIV). The video lecture introduces the ultrasound machine, learning objectives, knobology, and the ultrasound probes. The SIV, on the other hand, shows the psychomotor skills involved in performing FAST, and interpretation of the images. These videos were developed by a team from our department of emergency medicine. The videos were then edited using VideoPad Video Editor V10.04 (NCH Software, Canberra Australia). Simulation of the FAST was performed on a simulated patient. The same content was also prepared in PowerPoint slides for the classroom learning group. Both learning material were validated by senior lecturers and emergency physicians from the university hospital who are trainers for the FAST course in emergency medicine faculty.

#### Assessment Tools

The questionnaire evaluates the participants' socio-demography, confidence in performing FAST, and preference of learning FAST. For the confidence, a 5-point Likert scale was used whereas a close ended question was for the preferences. The content of the questionnaire was validated by senior emergency physicians. Internal consistency reliability testing was done using statistical analysis and the alpha score obtained for our questionnaire was 0.75. The OSCE checklist used for assessing the participants' proficiency in performing FAST was created based on standard FAST protocol. It was reviewed and validated by an expert panel comprising of two emergency physicians.

#### Participant Enrolment

An invitation to participate in this study was forwarded to all house officers (*n* = 235) in HCTM from 6 departments (internal medicine, surgery, orthopedic, pediatric, and anaesthesiology). Those that agreed to join the study were given instructions via email on how to proceed with the study. Those who undergone formal ultrasound training for FAST or abdominal ultrasound or who did not give consent were excluded. Selected participants were then randomized into two parallel groups: the SIV group (intervention) and, the CTM group (control). The randomization was done using table of random numbers. Allocation of the participants into the 2 groups was done via a sealed envelope by the investigator (LK).

#### Study Protocol

Participants in the intervention group was given the video on FAST after they were assigned to their smaller groups. They were advised to go through the video from the beginning until the end at least once before they proceed with hands-on training. They were allocated 4 h in the training room to learn and practice using the video given on all five simulated patients for that day. There were no facilitators assigned to guide the intervention group participants. The intervention duration was decided based on our literature review and actual courses conducted for FAST (4, 18).

Meanwhile, the control group received a lecture delivered by an emergency medicine resident, followed by hands-on training on 5 simulated patients. Image acquisition and interpretation were guided by facilitators. The participants must perform on all 5 simulated patients. The total time allocated for teaching and practice was 4 h.

Assessment was done on the same day as the practice using the validated OSCE and questionnaire. Two stations were prepared. Station 1 was to test their ability to obtain the four images in FAST (the hepatorenal view, splenorenal view, pelvic view, and subxiphoid view) while station 2 was to test their ability to interpret the FAST images (normal and abnormal). The test was performed on the same stimulated patients that were used for the hands-on training. The assessors were blinded from the teaching method that the participants received. Each station was allocated 10 min for the participants to complete their tasks. Upon completion of the OSCE, the participants were given a post-test questionnaire to complete and allowed home. The participants on the first day were strongly advised not to share any information with their peers that were attending the next day. Participants for both days were mixed of the intervention and control groups.

### Outcome Measurement

The primary outcome measures were the ability of the participants to obtain the four images in FAST, and their ability to interpret the images with normal and abnormal findings. Both outcomes were measured based on the OSCE checklist. Secondary outcome measures were the participants' learning preference and the confidence level to perform FAST. The scores were based on a five-point Likert scale. The maximum score for the OSCE was 32 marks, whereas for image acquisition it was 14 marks.

### Sample Size

The sample size was calculated based on the non-inferiority trials with continuous variables (Sealed Envelope Ltd, 2012), using a significance level of 5%, power of 90%, standard deviation, σ of 9.3 and a non-inferiority limit δ of 10%. This gave a sample size of 15 per arm. In order to adjust for an estimated dropout rate of 20%, we aimed to recruit 18 participants per arm. The standard deviation was based on results from a previous study on video-based learning (12). The non-inferiority limit was based on similar studies (8, 12).

### Calibration of Assessors

The OSCE assessors comprised emergency physicians and senior emergency medicine residents trained and certified to perform FAST in their daily practice. They were invited to join the inter-rater calibration session conducted prior to the assessment. The examiners were briefed on the study objective, their roles and the OSCE checklist. After the briefing, they were shown images and video clips of other medical interns performing FAST and asked to evaluate the videos based on the checklist given. Upon completion, the results and crucial steps were discussed to clear any confusion and ensure unanimity among their evaluation during data collection. The intra-class correlation (ICC) score calculated for all five assessors was 0.69 with confidence interval set at 95%.

### Statistical Analysis

Statistical analysis was carried out using Statistical Packages for Social Science (SPSS), version 22.0 (IBM Armonk, NY). The data obtained from the questionnaire were analyzed using descriptive statistics. Mann Whitney *U*-test was applied for questions with the Likert scale. We defined a *p*-value of <0.05 as statistically significant. Normality of the variable distributions was determined using Shapiro-Wilk test. Descriptive analyses were done on the OSCE results and expressed as mean and standard deviation. The scores from both groups were compared using independent Students *t*-test and Mann Whitney *U*-test. To determine the non-inferiority of the video intervention group, the results were plotted in a graph to compare the upper bound of the confidence interval to the non-inferiority margin. The non-inferiority margin was set at 10% based on previous studies to compare classroom-based learning with video-based learning (8, 12).

## Results

**Figure 1:**
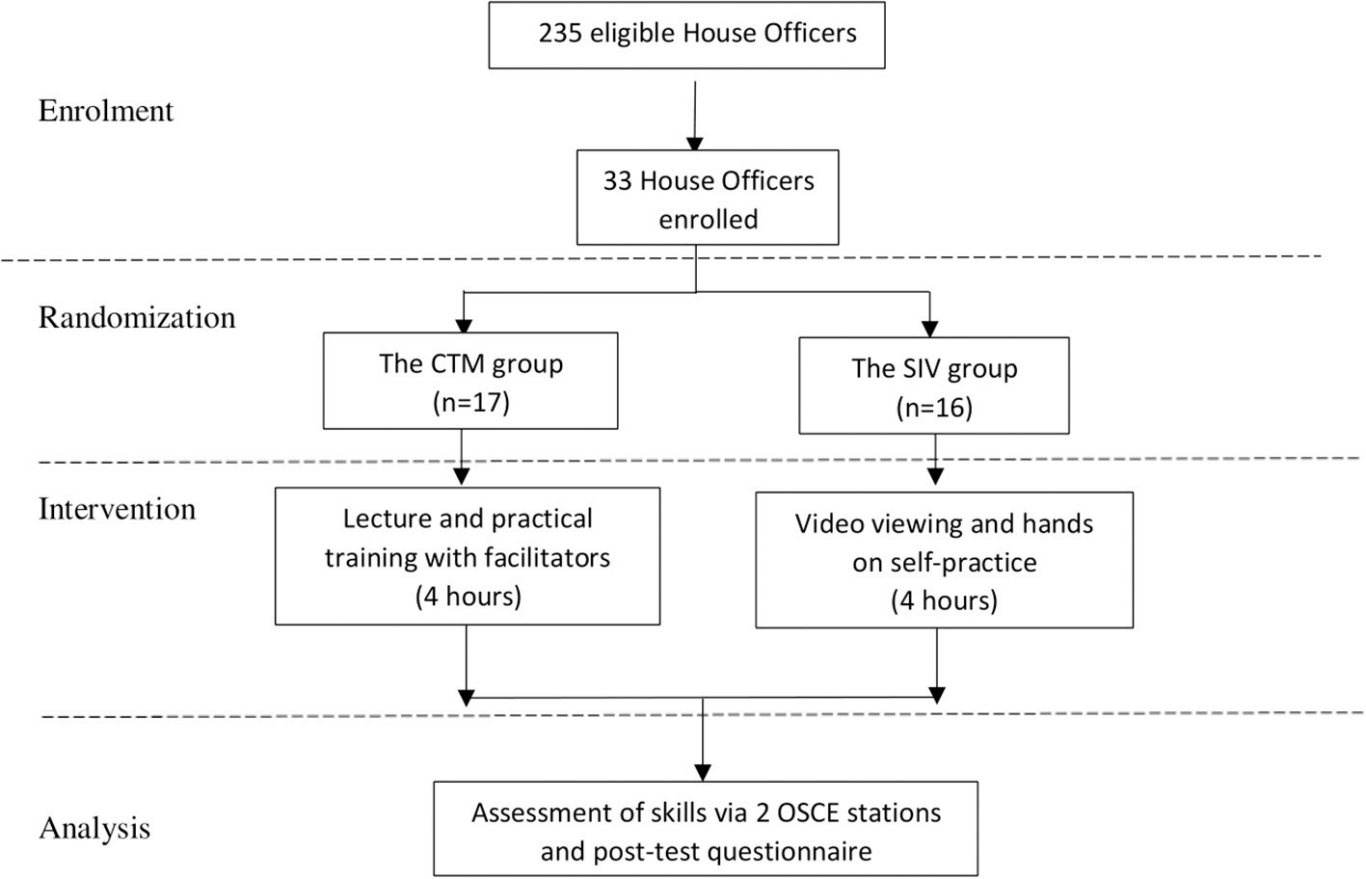
Study flow chart according to CONSORT guidelines.

Out of 235 potential recruits, 38 house officers volunteered to participate. Only 33 were enrolled based on inclusion and exclusion criteria of this study. Sixteen were in the intervention and 17 in the control groups (Figure 1). The participants were between 25 and 35 years of age. Four out of 33 participants (12%) had prior exposure to ultrasound courses covering obstetric ultrasound and vascular access but did not fulfill the exclusion criteria. None of our participants had prior learning on FAST or prior experience performing FAST. There were no significant demographic differences between these two groups with the *p* > 0.05 as shown in Table 1.

**Table 1:** Baseline characteristics of participants from both groups.

**Variable**	**SIV group** **(*N* = 16)**	**CTM group** **(*N* = 17)**	***p*-value**
	***n* (%)**	***n* (%)**	
Gender			
Male Female	8 (50) 8 (50)	6 (35) 11 (65)	0.491
Work experience			
1st year HO 2nd year HO	10 (63) 6 (37)	15 (88) 2 (12)	0.118
Prior experience with video learning			
Yes No	15 (94) 1 (6)	12 (71) 5 (29)	0.175
Prior exposure to ultrasound training			
Yes NO	2 (13) 14 (87)	2 (12) 15 (88)	1.000

*SIV, self-instructional video; CTM, conventional training method; HO, House-officer*.

**Figure 2:**
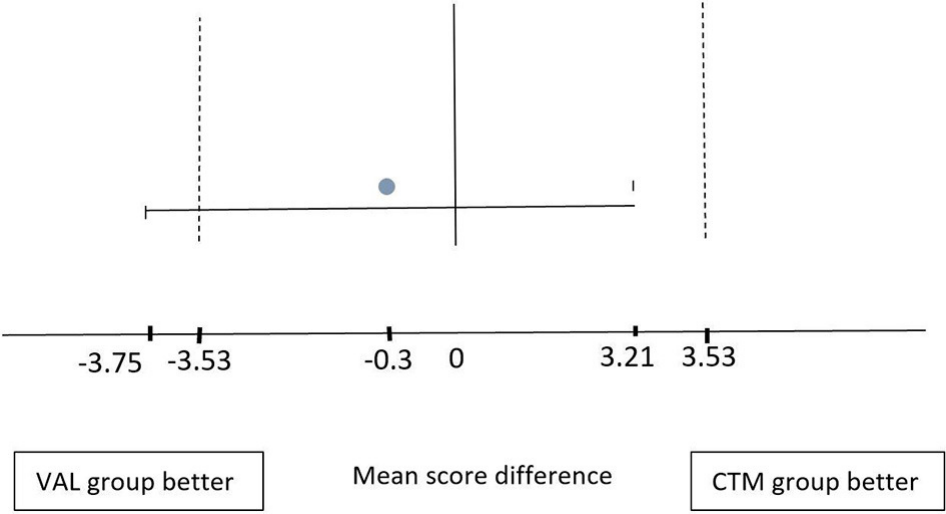
Mean-score difference graph demonstrating that the 10% confidence interval (CI) mean score of the control group is between −3.53 and 3.53 (dotted line). The absolute difference of the mean score between both groups is negative −0.3 (gray bullet). The intervention group mean score CI is between −3.75 and 3.21 (continuous line). The upper bound of CI is within the 10% difference margin. This shows the non-inferiority of the OSCE score of the intervention group compared to control.

For FAST performance in image acquisition (Table 2), the control group mean score was 25.6 (SD = 2.3) while the intervention group scored 25.3 (SD = 5.3). However, there was no significant difference between groups (*p* > 0.05). In image interpretation, the intervention group mean score was 10.3 (SD = 1.7) and 9.8 (SD = 1.7) for the control with no statistically significant difference (*p* > 0.05). Total mean scores for the intervention and control groups were 35.6 (SD = 5.9) and 35.3 (SD = 3.4), respectively. The absolute difference between the mean score of the two groups was 0.3 (CI = −3.75 to 3.21, *p* = 0.871), hence according to Figure 2, it only proves non-inferiority but not superiority between the two groups.

**Table 2:** Assessment of OSCE results after intervention.

	**SIV group** **(*n* = 16)**	**CTM group** **(*n* = 17)**	***p*-value^b^**	**Mean score difference**
Mean Station 1^a^ performing FAST (SD) [Total marks 31]	25.3 (5.3)	25.6 (2.3)	0.845	0.3
Mean Station 2^a^ interpret images (SD) [Total marks 14]	10.3 (1.7)	9.8 (1.7)	0.359	0.5
Mean Total OSCE score^a^ (SD) [Total marks 45]	35.6 (5.9)	35.3 (3.4)	0.871	0.3

*SIV, self-instructional video; CTM, conventional training method; OSCE, objective structured clinical examination; FAST, focused assessment with sonography in trauma*.

a *Data reported as mean score (standard deviation)*.

b *P-value is reported with 95% confidence interval*.

In terms of learning method preference, 22 (67%) participants favored the control group. The details of findings in the control and intervention groups are shown in Table 3.

**Table 3:** Summary of response from participants regarding their confidence level and preferred learning method.

	**SIV group** **(*n* = 16)** ***n* (%)**	**CTM group** **(*n* = 17)** ***n* (%)**	***p*-value**
Preferred learning method^c^			
Classroom Video Both Not applicable^a^	12 (80) 2 (13) 1 (7) 1	10 (84) 1 (8) 1 (8) 5	1.000
Possibility to learn skills through video^c^			
Yes No	15 (94) 0^b^	14 (82) 3 (19)	0.229
My knowledge level on FAST is adequate after training^d^			
Disagree Neutral Agree Strongly agree	0 5 (31) 11 (69) 0	0 2 (12) 9 (53) 6 (35)	**0.014**
I am confident in performing FAST after training^d^			
Disagree Neutral Agree Strongly agree	1 (6) 6 (38) 8 (50) 1 (6)	0 0 11 (65) 6 (35)	**0.002**
I am confident in interpreting images obtained in FAST^d^			
Disagree Neutral Agree Strongly agree	2 (13) 8 (50) 5 (31) 1 (6)	0 2 (12) 10 (59) 5 (29)	**0.002**
I will perform FAST on real patients^d^			
Disagree Neutral Agree Strongly agree	0 4 (25) 7 (44) 5 (31)	0 0 8 (47) 9 (53)	0.720

a *5 participants did not answer this question because they never had video learning and 1 missing data, these 6 samples were not included in the analysis*.

b *1 missing data*.

c *Fisher Exact test*.

d *Mann Whitney U-test*.

All participants in the control group were confidence to perform FAST as compared to only 9 (56%) in the intervention group. In the control group, the majority (15, 88%) were confident to interpret the images obtained while performing FAST, whereas only 6 (38%) in the intervention group expressed confidence. The differences in confidence to perform FAST and to interpret images between the two groups were statistically significant (*p* < 0.05) (Table 3).

All participants (100%) in the control group and 12 (75%) in the intervention group were willing to perform FAST on real patients, however this difference was not statistically significant (*p* > 0.05) (Table 3).

## Discussion

Video learning has been used in various medical fields to teach clinical procedures and impart knowledge with good outcomes (19, 20). Our findings reaffirm previous reports. In this study, the intervention group showed similar efficacy compared to the control group for learning FAST. This leads us to conclude that SIV made it possible for house officers to learn FAST, indicated by non-inferior marks on their performance as compared to the control group. The OSCE score in both learning groups was within the 10% difference margin pre-specified before the intervention. In fact, the marks in the intervention group were higher than the control group, although the difference was not statistically significant. The comparable OSCE results between these two groups shows that, despite a shorter course duration and the absence of on-site instructor guidance, the intervention group was still capable of performing within the allocated time.

Our evidence is in keeping with results obtained from various studies that compared traditional classroom learning with video learning of sonographic skills (21, 22), indicating that it is possible for medical instructors to incorporate video learning into their curriculum. Many studies have clearly demonstrated that modern teaching techniques facilitate training and student achievement (23, 24). Students are given more responsibility to ensure their learning is complete and they share the instructors' burden. They are provided the opportunity to do this at their own pace and in the environment of their choice (10, 12). This is beneficial especially for house officers with long working hours and inflexible schedules. A study by Woodham et al. (25) reported that both trainees and tutors felt that video learning delayed the learning process. Conversely, in our study, the two groups underwent learning within the same time frame and produced similar results, which means that SIV does not necessarily prolong the learning process.

In the survey among our participants, 82% stated their preference for CTM over SIV although they had prior experience with video learning for other skills during their undergraduate years. Even among our intervention SIV group, 75% participants still preferred CTM. Interestingly, 92% of those with video learning experience agreed that it is possible to learn skills using SIV although the majority preferred CTM.

Based on these findings, we present three important points. Firstly, SIV is ubiquitous (26). This is implied from the widespread availability of the internet, hence all information and resources are easily accessible through gadgets such as computers and smartphones. Our participants were house officers who are mostly in their late twenties, hence they are most likely to be familiar with the use of the internet to gain information, being digital natives. Furthermore, many medical universities have embedded video learning or e-learning into their curriculum (14). Our participants were likely to have been exposed to these alternative learning methods as medical undergraduates.

Secondly, performing FAST is a complex learning procedure. Despite that, SIV was capable of imparting psychomotor skills to the participants as shown by the marks obtained, which were better than the control group albeit statistically insignificant. This evidence was previously supported by the advantage of SIV in teaching the complex skills of endotracheal intubation among medical students (27).

Thirdly despite being receptive to video learning, most participants prefer CTM for gaining new skills. Soon et al. (8) postulated that their participants favored CTM because they were mostly of older age with a median of 7 years' work experience. The actual age range was, however, not stated. Interestingly, our findings were different from theirs. Our participants were of a younger age with only 1–2 years' work experience, yet their preference was CTM. We attributed this finding to the lack of confidence in SIV.

Based on our questionnaire results, the intervention group was less confident in performing FAST and interpreting the images as compared to the control group. However, we did not reveal the assessment outcome of both groups to the participants. We did not explore the possibility of them changing their perception if they had known the primary outcome of this study. Similarly, we did not explore the reason why our participants preferred classroom learning over video learning. We postulated that it might be due to lack of feedback from an instructor, making the intervention group participants unsure if they were performing correctly. Students often use feedback they received in order to guide further learning direction and effort, especially from their tutors or instructors (28). As other authors have observed, the use of feedback correlates positively with exam scores (29). Sekiguchi et al. (11) also emphasized the importance of hands-on training with supervision because their participants scored lower than expected prior to training with supervision. This differs from the findings in other studies. Soon et al. (8) reported that the comfort level was similar in both intervention arms post-test. Back et al. (7), reported a significant increase in confidence score after ultrasound video tutorial. However, their study did not have a comparison arm. We looked into the literature for other obstacles that might be the reason for our participants' preference for CTM over SIV. In a qualitative study on teaching clinical skills to nurses, students highlighted the need to talk to a tutor or instructor especially when learning a new skill (29, 30).

Video learning is largely dependent on the students themselves. They need to analyze and extract information from the video themselves, which may make the learning process more challenging compared to classroom learning by a tutor (29, 30). Overall, most participants in our study were receptive toward video learning and agreed that video was a good learning tool especially for skills and procedures. There are weaknesses in video learning, including the need for customization to suit students' needs (31). For example, our video had visual and audio prompts, and labeling on the images, but no closed captioning. The use of closed captioning might improve audience understanding of the content.

The strength of our study is the full utilization of the video material to test its effectiveness. Participants were not given any other guidance to learn FAST until after the data collection was completed. We standardized the learning conditions between the intervention and control groups, including the learning material, time for learning the material given, the venue, and the ultrasound scanner, in order to allow a fair comparison. We selected participants who are not skilled in performing FAST in order to get a homogenous sample. This allowed us to better evaluate the effect of the learning methods used with fewer confounding factors.

### Limitations

There were several limitations in this study. Firstly, the participants' satisfaction level, perception of SIV for training of sonographic skills, and their reasons for preference for the learning method, were all not explored. A mixed methods study might reveal a better understanding of the participants' response. Further study to glean such information will enable medical instructors to better cater for students' needs. Secondly, the small sample size was contributed by difficulties in enrolment of house officers into the study. We found the majority of them had already learned FAST through on-job-training. Another contributing factor was the limited time available among house officers and the inflexibility of their work schedule. Future studies with a larger sample size are needed to provide a more generalizable interpretation of results. Finally, this study used an asynchronous non-feedback video teaching approach, therefore participants could not air or voice their queries or concern.

## Conclusion

Our study showed that SIV is as effective as face-to-face classroom method in teaching house officers to perform FAST. However, this learning tool needs improvement in order to promote house officers' confidence and preference. Perhaps the inclusion of a feedback session after going through the SIV would help improve their confidence in performing FAST and interpreting the images.

## Data Availability Statement

The raw data supporting the conclusions of this article will be made available by the authors, without undue reservation.

## Ethics Statement

The studies involving human participants were reviewed and approved by Medical Ethic and Research Committee of Universiti Kebangsaan Malaysia (Ethic Approval no: JEP-2019-269. The patients/participants provided their written informed consent to participate in this study.

## Author Contributions

IMS involved in the study concept, data interpretation, and writing. MHI involved in the study concept, data collection, data interpretation, and writing. KL involved in data collection, data interpretation, and writing. MJJ involved in conceptual and data interpretation. All authors contributed to the article and approved the submitted version.

## Conflict of Interest

The authors declare that the research was conducted in the absence of any commercial or financial relationships that could be construed as a potential conflict of interest.

## Publisher's Note

All claims expressed in this article are solely those of the authors and do not necessarily represent those of their affiliated organizations, or those of the publisher, the editors and the reviewers. Any product that may be evaluated in this article, or claim that may be made by its manufacturer, is not guaranteed or endorsed by the publisher.
